# Supplementary material for: The global importance and interplay of colour-based protective and thermoregulatory functions in frogs
Source: Nat Commun. 2023 Dec 19;14:8117. doi: 10.1038/s41467-023-43729-7 (PMC10730650; doi:10.1038/s41467-023-43729-7)
Supplement: Supplementary file 1 — Supplementary Information [file 41467_2023_43729_MOESM1_ESM.pdf]

SUPPLEMENTARY INFORMATION for

The global importance and interplay of colour-based protective and thermoregulatory functions in frogs

Ricarda Laumeier[1,2]*, Martin Brändle[1], Mark-Oliver Rödel[3], Stefan Brunzel[2], Roland Brandl[1], Stefan Pinkert[4,5]

[1]Department of Animal Ecology, Faculty of Biology, Philipps-Universität Marburg, Karl-von-Frisch-Straße 8, 35043 Marburg, Germany

[2]Department of Biodiversity and Species Conservation, Faculty of Landscape Architecture, Horticulture and Forestry, University of Applied Science Erfurt, Leipziger Straße 77, 99085 Erfurt, Germany

[3]Department of Evolutionary Diversity Dynamics, Museum für Naturkunde – Leibniz Institute for Evolution and Biodiversity Science, Invalidenstraße 43, 10115 Berlin, Germany

[4]Department of Ecology and Evolutionary Biology, Yale University, 165 Prospect Street, 06511 New Haven, CT, USA

[5]Department of Conservation Ecology, Faculty of Biology, Philipps-Universität Marburg, Karl-von-Frisch-Straße 8, 35043 Marburg, Germany

CORRESPONDING AUTHOR

*Ricarda Laumeier (ricardalaumeier@gmail.com)

Supplementary Table 1. Colour lightness–environment relationships of diurnal and nocturnal anurans. Multiple linear regressions of colour lightness and interactions of EVI, mean annual temperature, elevation and annual UVB separated with activity time (D = diurnal, N = nocturnal, D/N = diurnal and nocturnal, n = 820 species).

Interaction		Slope ± SE	t	P	R^2
Mean annual EVI	D	–5.50 ± 8.74	–0.63	0.529	0.03
	N	–4.91 ± 2.93	–1.68	0.094	
	D/N	–11.00 ± 3.37	–3.26	0.001	
Mean annual temperature	D	11.41 ± 22.02	0.52	0.605	
	N	26.78 ± 11.53	2.32	0.021	
	D/N	26.98 ± 12.70	2.13	0.034	
Elevation	D	–0.23 ± 5.27	–0.04	0.965	
	N	3.82 ± 3.17	1.20	0.229	
	D/N	3.66 ± 3.86	0.95	0.343	
Mean annual UVB	D	–2.04 ± 12.62	–0.16	0.872	
	N	–6.30 ± 6.71	–0.94	0.348	
	D/N	–10.04 ± 8.04	–1.25	0.212	

Supplementary Table 2. Literature sources of species images used for quantifying colour lightness.

Literature	Year	Region/Country	Continent	Species
Dodd Jr, C. K., *Frogs of the United States and Canada - 2 volumes* Johns Hopkins University Press, Baltimore	2013	Canada and USA	North America	105
Speybroeck, J. B. B., Beukema, W., Bok, B., & van der Voort, J., *Field Guide to the Amphibians and Reptiles of Britain and Europe*. Bloomsbury Wildlife, London.	2018	Europe	Europe	46
Channing, A., & Rödel, M.-O., *Field guide to the frogs & other amphibians of Africa* Struik Nature, Cape Town.	2019	Africa	Africa	788
Glaw, F., & Vences, M. A., *Field Guide to the amphibians and reptiles of Madagascar. 3rd ed.,* Vences & Glaw Verlag, Cologne.	2007	Madagascar	Africa	239
Gururaja, K., *Pictorial guide to frogs and toads of the Western Ghats.* Gubbi Labs, Karnataka.	2012	Western Ghats, India	Asia	73
Gururaja, K., & Ramachandra, T., *Anuran diversity and distribution in Dandeli Anshi Tiger Reserve.* Energy & Wetlands. Research Group Centre for Ecological Sciences Indian Institute of Science Bangalore.	2012	Anshi Tiger Reserve, India	Asia	2
Fei, L., *Atlas of amphibians of China (field edition).* Zhongyuan Publishing and Media Group, Henan Science and Technology Press.	2020	China	Asia	357
Alhadi, F., Kaprawi, F., Hamidy, A., & Kirschey, T., *Amfibi Pulau Jawa - Panduan Bergambar dan Identifikasi.* Perkumpulan Amfibi Reptil Sumatera & NABU, Jakarta.	2021	Jawa, Indonesia	Asia	32
Inger, R. F., Stuebing, R. B., Grafe, U., & Dehling, M. A., *Field guide to the frogs of Borneo. 3rd ed.,* Natural History Publications (Borneo), Kota Kinabalu.	2017	Borneo, Indonesia Malaysia, Brunei	Asia	160
Tyler, M. J., & Knight, F., *Field guide to the frogs of Australia. 2nd ed.,* CSIRO Publishing, Clayton South.	2020	Australia	Australia	263

McCranie, J. R., & Wilson, L. D., *The amphibians of Honduras*. Society for the Study of Amphibians and Reptiles, Ithaca.	2002	Honduras	South America	47
Leenders, T., *Amphibians of Costa Rica: A field guide.* Zona Tropical Publication, Comstock Publishing Association, Ithaca.	2016	Costa Rica	Central America	146
Lescure, J., & Marty, C., *Atlas des amphibiens de Guyane*. Muséum national d'Histoire naturelle, Patrimoines naturels.	2000	Guyana	South America	19
Ouboter, P. E., & Jairam, R., *Amphibians of Suriname*. Brill, Leiden.	2012	Suriname	South America	46
Ron, S. R., Merino-Viteri, A., & Ortiz, D., *Anfibios del Ecuador - guía dinámica de especies Versión 2020.0.* Museo de Zoología, Pontificia Universidad Católica del Ecuador.	2021	Ecuador	South America	607
Kahn, T. R., la Marca, E., Lötters, S., Brown, J. L., Twomey, E., & Amézquita, A., *Aposematic Poison Frogs (Dendrobatidae) of the Andean Countries: Colombia, Bolivia, Ecuador, Peru and Venezuela*. Conservation International, Arlington.	2016	Andean Countries	South America	52
Bartlett, R. D., & Bartlett, P., *Reptiles and amphibians of the Amazon: An ecotourist's guide*. University Press of Florida, Gainsville	2003	Amazon	South America	27
Lima, A. P., Magnusson, W. E., Menin, M., Erdtmann, L. K., Rodrigues, D. de J., Keller, C. K., & Hödl, W., *Guia de Sapos da Reserva Adolpho Ducke: Amazônia Central*. Attema Design Editorial, Manaus.	2005	Amazon, Reserva Adolpho Ducke, Brazil	South America	4
Freitas, M. A., *Herpetofauna no nordeste Brasileiro - guia de Campo*. Technical Books Editora, Rio de Janeiro.	2015	northeast Brazil	South America	80
Ávila, R. W., Morais, D. H., Maffei, F., Pansonato, A., Kawashita-Ribeiro, R. A., Rodrigues, D. J., & Strüssmann, C., *Herpetofauna de Mato Grosso Vol. I - Anfíbios*. Editora CRV, Curitiba	2021	Mato Grosso, Brazil	South America	74

Maffei, F., Ubaid, F., & Jim, J., *Anfíbios - Fazenda Rio Claro, Lençóis Paulista, SP*. Canal 6 editora, Bauru.	2011	Rio Claro, Brazil	South America	3
Haddad, C. F. B., Toledo, L. F., Prado, C. P. A., Loebmann, D., Gasparini, J. L., & Sazima, I., *Guia dos Anfíbios da Mata Atlântica - Diversidade e Biologia*. Anolis Books, São Paulo.	2013	Mata Atlantica, Brazil	South America	451
Maneyro, R., Loebmann, D., Tozetti, A., & Fonte, L. F. M., *Anfíbios das planícies costeiras do extremo sul do Brasil e Uruguai*.Anolis Books, São Paulo.	2017	southern Brazil, Uruguay	South America	10
Uetanabaro, M., Prado, C. P. de A., Rodrigues, D. de J., Gordo, M., & Campos, Z., *Guia de campo dos anuros do Pantanal e planaltos de entorno*. Editora UFMS, Campo Grande.	2008	Pantanal, Brazil	South America	20
Charrier, A., *Guía de Campo Anfibios de los Bosques de la Zona Centro Sur y Patagonia de Chile*. Corma Chile.	2019	Chile	South America	43
Cei, J. M., *Batracios de Chile*. Ediciones de la Universidad de Chile, Santiago de Chile.	1962	Chile	South America	2
Cei, J. M., *Amphibians of Argentina*. Monitore zoologico italiano (N.S.), Monografia 2.	1980	Argentina	South America	49

Supplementary Table 3. Data coverage per family. Coverage of colour lightness and distributional data of all anuran families.

Family	Total species	Species with colour values	Species in distribution	% coverage colour	% coverage distribution
Allophrynidae	3	1	3	33	100
Ascaphidae	2	1	2	50	100
Nasikabatrachidae	2	1	2	50	100
Rhinophrynidae	1	1	1	100	100
Pelodytidae	5	2	5	40	100
Micrixalidae	24	3	24	13	100
Ranixalidae	17	3	17	18	100
Rhinodermatidae	3	3	3	100	100
Calyptocephalellidae	5	4	5	8	100
Pelobatidae	6	4	6	67	100
Telmatobiidae	63	4	63	6	100
Bombinatoridae	8	5	8	63	100
Ceratobatrachidae	97	5	97	5	100
Odontobatrachidae	5	5	5	100	100
Conrauidae	6	6	6	100	100
Heleophrynidae	7	6	7	85	100
Hemisotidae	9	7	9	78	100
Nyctibatrachidae	39	7	39	18	100
Scaphiopodidae	7	7	7	100	100
Alytidae	12	10	12	83	100
Batrachylidae	14	11	14	79	100
Ceratophryidae	12	11	12	92	100
Petropedetidae	13	11	13	85	100
Eleutherodactylidae	225	15	225	7	100
Aromobatidae	124	16	124	13	100
Odontophrynidae	53	18	53	34	100
Alsodidae	26	21	26	81	100
Cycloramphidae	36	22	36	61	100
Brachycephalidae	72	29	72	40	100
Hylodidae	47	30	47	64	100
Hemiphractidae	111	31	111	28	100
Brevicipitidae	36	33	36	92	100
Pipidae	41	38	41	93	100
Limnodynastidae	43	39	43	91	100
Phyllomedusidae	66	41	66	62	100
Ptychadenidae	59	47	59	80	100
Dicroglossidae	213	58	213	27	100
Centrolenidae	155	60	155	39	100
Phrynobatrachidae	91	61	91	67	100
Pelodryadidae	212	68	212	32	100
Myobatrachidae	89	69	89	78	100
Pyxicephalidae	84	73	84	87	100
Dendrobatidae	197	108	197	55	100
Megophryidae	231	113	231	49	100

Rhacophoridae	436	114	436	26	100
Arthroleptidae	150	125	150	83	100
Leptodactylidae	206	133	206	65	100
Hyperoliidae	226	166	226	73	100
Mantellidae	228	167	228	73	100
Microhylidae	713	171	713	24	100
Ranidae	396	183	396	46	100
Craugastoridae	825	245	825	30	100
Bufonidae	617	286	617	46	100
Hylidae	716	361	716	50	100
Leiopelmatidae	4	0	4	0	100
Sooglossidae	4	0	4	0	100

Note: Our initial colour lightness data included 51 species without distribution data. These were almost exclusively species from a source for Ecuadorian anurans (Ron et al. 2021) and noted as newly described species.

Supplementary Table 4. RGB values of the colour wheel.

Colour	R	G	B
Algaegreen10	205	204	184
Algaegreen100	127	123	0
Algaegreen40	177	177	107
Algaegreen70	154	153	45
Black10	255	255	255
Black100	24	24	24
Black40	177	177	177
Black70	102	102	102
Blue10	230	230	254
Blue100	0	0	254
Blue40	154	153	255
Blue70	77	76	255
Brown10	204	194	184
Brown100	123	61	0
Brown40	176	142	107
Brown70	152	99	45
Burgundy10	203	184	204
Burgundy100	126	0	126
Burgundy40	177	107	177
Burgundy70	153	45	154
Cyan10	231	255	255
Cyan100	1	255	255
Cyan40	153	255	255
Cyan70	77	255	255
Darkblue10	183	184	204
Darkblue100	0	0	126
Darkblue40	107	107	177
Darkblue70	44	45	153
Darkcyan10	159	177	177
Darkcyan100	0	126	125
Darkcyan40	107	177	177
Darkcyan70	45	153	153
Darkgreen10	183	200	181
Darkgreen100	0	126	0
Darkgreen40	107	177	107
Darkgreen70	45	153	44
Darkred10	204	184	185
Darkred100	128	0	1
Darkred40	177	107	107
Darkred70	153	45	45
Elder10	250	230	255
Elder100	210	0	255

Elder40	235	153	255
Elder70	224	77	254
Green10	233	255	234
Green100	0	255	1
Green40	153	255	153
Green70	76	255	76
Magenta10	255	230	254
Magenta100	255	0	254
Magenta40	255	153	255
Magenta70	255	77	255
Orange10	255	244	230
Orange100	254	153	0
Orange40	255	210	153
Orange70	255	177	77
Red10	255	230	230
Red100	255	0	0
Red40	255	152	153
Red70	255	77	77
Sky10	230	255	251
Sky100	0	255	211
Sky40	152	255	236
Sky70	77	254	225
Warmgrey10	205	192	184
Warmgrey100	51	47	44
Warmgrey40	153	144	137
Warmgrey70	103	95	92
Yellow10	255	255	229
Yellow100	255	255	1
Yellow40	255	251	152
Yellow70	255	255	77

Supplementary Table 5. Indirect and direct effects of environmental predictors on the colour lightness of anuran assemblages from structural equation modelling (n = 16,686 assemblages). Results from multiple linear mixed effects models (predictor slopes ± SE, standardised estimates, P, R^2) that included a random slope effect for biogeographical realm identity. These models were fitted as component models in a structural equation modelling framework with functions of the R package piecewiseSEM and lme. Significance of the correlation coefficients was tested using two-sided t-test. Causal relationships between environmental predictors are fitted based on theoretical assumptions. Only for the relationship between tree cover and productivity (EVI) an unresolved causality was assumed. The relationships between environmental factors and colour lightness were included following the predictions of the thermal melanism hypothesis (colour lightness decreases with decreasing temperature and increasing elevation), the Gloger's rule (colour lightness increases with productivity), and the UBV-protection hypothesis (colour lightness increases with decreasing UVB radiation). In addition, the model assumed a potential effect of tree cover on colour lightness assuming darker species are better camouflaged in regions with greater tree coverage (more shaded).

Response	Predictor	Slope ± SE	t	P	Marginal R^2	Conditional R^2
Colour lightness	MAT	20.521 ± 0.485	42.338	$< 10^{-16}$	0.26	0.52
	Elevation	4.599 ± 0.178	25.864	$< 10^{-16}$		
	EVI	–2.744 ± 0.192	–14.283	$< 10^{-16}$		
	Tree cover	–0.080 ± 0.161	–0.497	6.20×10^{-1}		
	UVB	–7.425 ± 0.424	–17.524	$< 10^{-16}$		
EVI	MAT	0.633 ± 0.015	41.359	$< 10^{-16}$	0.11	0.45
	UBV	–0.289 ± 0.014	–20.820	$< 10^{-16}$		
UVB	Elevation	0.174 ± 0.004	45.763	$< 10^{-16}$	0.04	0.73
MAT	Elevation	–0.303 ± 0.002	–168.389	$< 10^{-16}$	0.91	0.93
	UVB	0.782 ± 0.004	223.343	$< 10^{-16}$		
Tree cover	MAT	–0.574 ± 0.011	–52.164	$< 10^{-16}$	0.45	0.60
	EVI	0.785 ± 0.007	112.186	$< 10^{-16}$		

Note: Tree cover does not directly affect colour lightness (P = 0.62). Coefficients of the unresolved causality included in the initial model indicated that tree cover is driven by productivity rather than the other way around (Std. estimate: 0.7026 > 0.6241), latter effect was subsequently removed from the final model to evaluate whole model completeness and performance. Fisher's C statistics indicated that this resolved model included all significant effects (Fisher's C = 3.03 with P = 0.805). Together this underlines that camouflage (tree cover) is not confounding the effects of productivity (EVI) on colour lightness in our analysis. Abbreviation: MAT = Mean annual temperature, EVI = Mean annual Enhanced Vegetation Index (productivity), UVB = Mean annual UVB, tree cover = Proportion of tree cover per grid. Marginal/conditional R^2 = Explained variance from model without/with random slope effect.

Supplementary Table 6. Single regression of chytridiomycosis severity and (raw) colour lightness for anuran families (n = 1392 species, R^2 = 0.40). Significance of the correlation coefficients was tested using two-sided t-test.

Family	Slope ± SE	t	P
Alsodidae	0.001 ± 0.006	0.18	n.s
Alytidae	0.002 ± 0.003	0.71	n.s
Aromobatidae	0.039 ± 0.007	5.91	<0.001
Arthroleptidae	–0.002 ± 0.001	–1.29	n.s
Bombinatoridae	–0.005 ± 0.005	–0.86	n.s
Brachycephalidae	0.010 ± 0.005	2.11	<0.05
Brevicipitidae	–0.004 ± 0.002	–2.40	<0.05
Bufonidae	0.005 ± 0.001	5.19	<0.001
Calyptocephalellidae	0.020 ± 0.010	2.04	<0.05
Centrolenidae	0.007 ± 0.001	5.03	<0.001
Ceratobatrachidae	–0.005 ± 0.004	–1.14	n.s
Conrauidae	–0.005 ± 0.009	–0.52	n.s
Craugastoridae	0.024 ± 0.002	12.63	<0.001
Cycloramphidae	0.032 ± 0.009	3.69	<0.001
Dendrobatidae	0.012 ± 0.003	3.87	<0.001
Dicroglossidae	–0.004 ± 0.001	–3.10	<0.01
Eleutherodactylidae	0.006 ± 0.003	1.60	n.s
Heleophrynidae	–0.003 ± 0.003	–1.23	n.s
Hemiphractidae	0.010 ± 0.003	3.38	0.001
Hemisotidae	–0.004 ± 0.004	–1.01	0.313
Hylidae	0.008 ± 0.001	7.53	<0.001
Hylodidae	0.019 ± 0.004	4.43	<0.001
Hyperoliidae	–0.003 ± 0.001	–3.10	<0.01
Leptodactylidae	0.009 ± 0.002	3.61	<0.001
Limnodynastidae	–0.002 ± 0.002	–0.73	n.s
Mantellidae	–0.004 ± 0.001	–4.17	<0.001
Megophryidae	–0.005 ± 0.001	–3.80	<0.001
Micrixalidae	–0.004 ± 0.004	–0.88	n.s
Microhylidae	–0.004 ± 0.001	–3.56	<0.001
Myobatrachidae	0.010 ± 0.002	5.97	<0.001
Nasikabatrachidae	–0.009 ± 0.016	–0.52	n.s
Nyctibatrachidae	–0.006 ± 0.004	–1.33	n.s
Odontobatrachidae	–0.005 ± 0.007	–0.74	n.s
Odontophrynidae	0.008 ± 0.011	0.76	n.s
Pelobatidae	–0.003 ± 0.004	–0.73	n.s
Pelodryadidae	0.008 ± 0.001	5.71	<0.001
Petropedetidae	–0.001 ± 0.004	–0.15	n.s
Phrynobatrachidae	0.001 ± 0.002	0.71	n.s
Phyllomedusidae	0.013 ± 0.003	4.92	<0.001
Pipidae	–0.004 ± 0.003	–1.31	n.s
Ptychadenidae	–0.003 ± 0.002	–2.11	<0.05
Pyxicephalidae	–0.003 ± 0.001	–3.26	<0.01
Ranidae	–0.002 ± 0.001	–2.50	<0.05
Ranixalidae	–0.004 ± 0.005	–0.86	n.s

Rhacophoridae	–0.003 ± 0.001	–3.80	<0.001
Rhinodermatidae	0.022 ± 0.005	4.86	<0.001
Telmatobiidae	0.033 ± 0.007	4.98	<0.001

Supplementary Table 7. Single regression of chytridiomycosis severity and productivity (Annual EVI) for anuran families (n = 1392 species, $R^2 = 0.46$). Significance of the correlation coefficients was tested using two-sided t-test.

**Family**	**Slope ± SE**	**t**	***P***
Alsodidae	–0.400 ± 0.921	–0.43	n.s
Alytidae	1.096 ± 0.680	1.61	n.s
Aromobatidae	2.240 ± 0.351	6.38	<0.001
Arthroleptidae	0.081 ± 0.119	0.68	n.s
Bombinatoridae	–0.125 ± 0.337	–0.37	n.s
Brachycephalidae	0.917 ± 0.354	2.59	<0.01
Brevicipitidae	–0.159 ± 0.189	–0.84	n.s
Bufonidae	0.545 ± 0.058	9.47	<0.001
Calyptocephalellidae	4.419 ± 1.811	2.44	<0.05
Centrolenidae	0.899 ± 0.129	6.98	<0.001
Ceratobatrachidae	–0.086 ± 0.265	–0.32	n.s
Conrauidae	–0.294 ± 1.082	–0.27	n.s
Craugastoridae	1.407 ± 0.083	16.91	<0.001
Cycloramphidae	1.669 ± 0.383	4.36	<0.001
Dendrobatidae	0.970 ± 0.176	5.51	<0.001
Dicroglossidae	–0.148 ± 0.093	–1.60	n.s
Eleutherodactylidae	0.798 ± 0.266	3.00	<0.01
Heleophrynidae	–0.385 ± 1.033	–0.37	n.s
Hemiphractidae	0.878 ± 0.215	4.09	<0.001
Hemisotidae	–0.222 ± 0.428	–0.52	n.s
Hylidae	0.967 ± 0.069	14.06	<0.001
Hylodidae	1.116 ± 0.215	5.19	<0.001
Hyperoliidae	–0.145 ± 0.100	–1.46	n.s
Leptodactylidae	0.822 ± 0.146	5.64	<0.001
Limnodynastidae	0.903 ± 0.410	2.20	<0.05
Mantellidae	–0.163 ± 0.058	–2.79	<0.01
Megophryidae	–0.145 ± 0.064	–2.25	<0.05
Micrixalidae	–0.183 ± 0397	–0.46	n.s
Microhylidae	–0.117 ± 0.062	–1.88	n.s
Myobatrachidae	1.835 ± 0.169	10.86	<0.001
Nasikabatrachidae	–0.176 ± 0.648	–0.27	n.s
Nyctibatrachidae	–0.176 ± 0.257	–0.69	n.s
Odontobatrachidae	–0.152 ± 0.396	–0.38	n.s
Odontophrynidae	0.560 ± 0.516	1.08	n.s
Pelobatidae	–1.593 ± 4.280	–0.37	n.s
Pelodryadidae	1.728 ± 0.192	8.98	<0.001
Petropedetidae	0.121 ± 0.360	0.34	n.s
Phrynobatrachidae	0.381 ± 0.166	2.29	<0.05
Phyllomedusidae	1.098 ± 0.176	6.25	<0.001
Pipidae	–0.166 ± 0.272	–0.61	n.s
Ptychadenidae	–0.221 ± 0.216	–1.02	n.s
Pyxicephalidae	–0.167 ± 0.174	–0.96	n.s
Ranidae	–0.076 ± 0.062	–1.23	n.s
Ranixalidae	–0.183 ± 0.392	–0.47	n.s

Rhacophoridae	–0.126 ± 0.053	–2.37	<0.05
Rhinodermatidae	4.263 ± 0.884	4.82	<0.001
Telmatobiidae	3.213 ± 0.695	4.63	<0.001

Supplementary Table 8. Single regression of the interaction between (raw) colour lightness and productivity (Annual EVI) on chytridiomycosis severity for anuran families (n = 1392 species, R^2 = 0.42). According to the predictions of the Gloger's rule lighter-coloured species in regions with a higher productivity (higher pathogen pressure) should be more susceptible to fungal or bacterial pathogens. Hence the interaction of both high colour lightness and EVI should positively affect severity. Significance of the correlation coefficients was tested using two-sided t-test.

**Family**	**Slope ± SE**	**t**	***P***
Alsodidae	–0.0040 ± 0.0103	–0.39	n.s
Alytidae	0.0091 ± 0.0059	1.55	n.s
Aromobatidae	0.0351 ± 0.0053	6.58	<0.001
Arthroleptidae	0.0003 ± 0.0011	0.29	n.s
Bombinatoridae	–0.0027 ± 0.0060	–0.46	n.s
Brachycephalidae	0.0077 ± 0.0030	2.58	<0.05
Brevicipitidae	–0.0016 ± 0.0018	–0.88	n.s
Bufonidae	0.0064 ± 0.0006	10.63	<0.001
Calyptocephalellidad	0.0557 ± 0.0235	2.37	<0.05
Centrolenidae	0.0061 ± 0.0009	6.54	<0.001
Ceratobatrachidae	–0.0013 ± 0.0037	–0.35	n.s
Conrauidae	–0.0038 ± 0.0124	–0.31	n.s
Craugastoridae	0.0168 ± 0.0011	15.86	<0.001
Cycloramphidae	0.0255 ± 0.0061	4.16	<0.001
Dendrobatidae	0.0098 ± 0.0020	4.84	<0.001
Dicroglossidae	–0.0019 ± 0.0010	–1.78	n.s
Eleutherodactylidae	0.0092 ± 0.0033	2.78	<0.01
Heleophrynidae	–0.0035 ± 0.0082	–0.42	n.s
Hemiphractidae	0.0080 ± 0.0021	3.92	<0.001
Hemisotidae	–0.0025 ± 0.0043	–0.59	n.s
Hylidae	0.0081 ± 0.0006	13.33	<0.001
Hylodidae	0.0137 ± 0.0027	5.00	<0.001
Hyperoliidae	–0.0012 ± 0.0007	–1.64	n.s
Leptodactylidae	0.0079 ± 0.0016	5.00	<0.001
Limnodynastidae	0.0094 ± 0.0044	2.13	<0.05
Mantellidae	–0.0019 ± 0.0006	–3.12	<0.01
Megophryidae	–0.0019 ± 0.0008	–2.47	<0.05
Micrixalidae	–0.0019 ± 0.0037	–0.51	n.s
Microhylidae	–0.0016 ± 0.0007	–2.25	<0.05
Myobatrachidae	0.0198 ± 0.0019	10.47	<0.001
Nasikabatrachidae	–0.0042 ± 0.0139	–0.31	n.s
Nyctibatrachidae	–0.0028 ± 0.0037	–0.76	n.s
Odontobatrachidae	–0.0021 ± 0.0048	–0.43	n.s
Odontophrynidae	0.0076 ± 0.0074	1.03	n.s
Pelobatidae	–0.0116 ± 0.0288	–0.40	n.s
Pelodryadidae	0.0110 ± 0.0014	7.85	<0.001
Petropedetidae	0.0010 ± 0.0036	0.28	n.s
Phrynobatrachidae	0.0043 ± 0.0016	2.65	<0.01
Phyllomedusidae	0.0098 ± 0.0017	5.93	<0.001

Pipidae	–0.0021 ± 0.0032	–0.67	n.s
Ptychadenidae	–0.0021 ± 0.0019	–1.12	n.s
Pyxicephalidae	–0.0014 ± 0.0014	–0.96	n.s
Ranidae	–0.0011 ± 0.0007	–1.69	n.s
Ranixalidae	–0.0020 ± 0.0040	–0.50	n.s
Rhacophoridae	–0.0013 ± 0.0005	–2.65	<0.01
Rhinodermatidae	0.0387 ± 0.0077	5.01	<0.001
Telmatobiidae	0.0330 ± 0.0069	4.75	<0.001

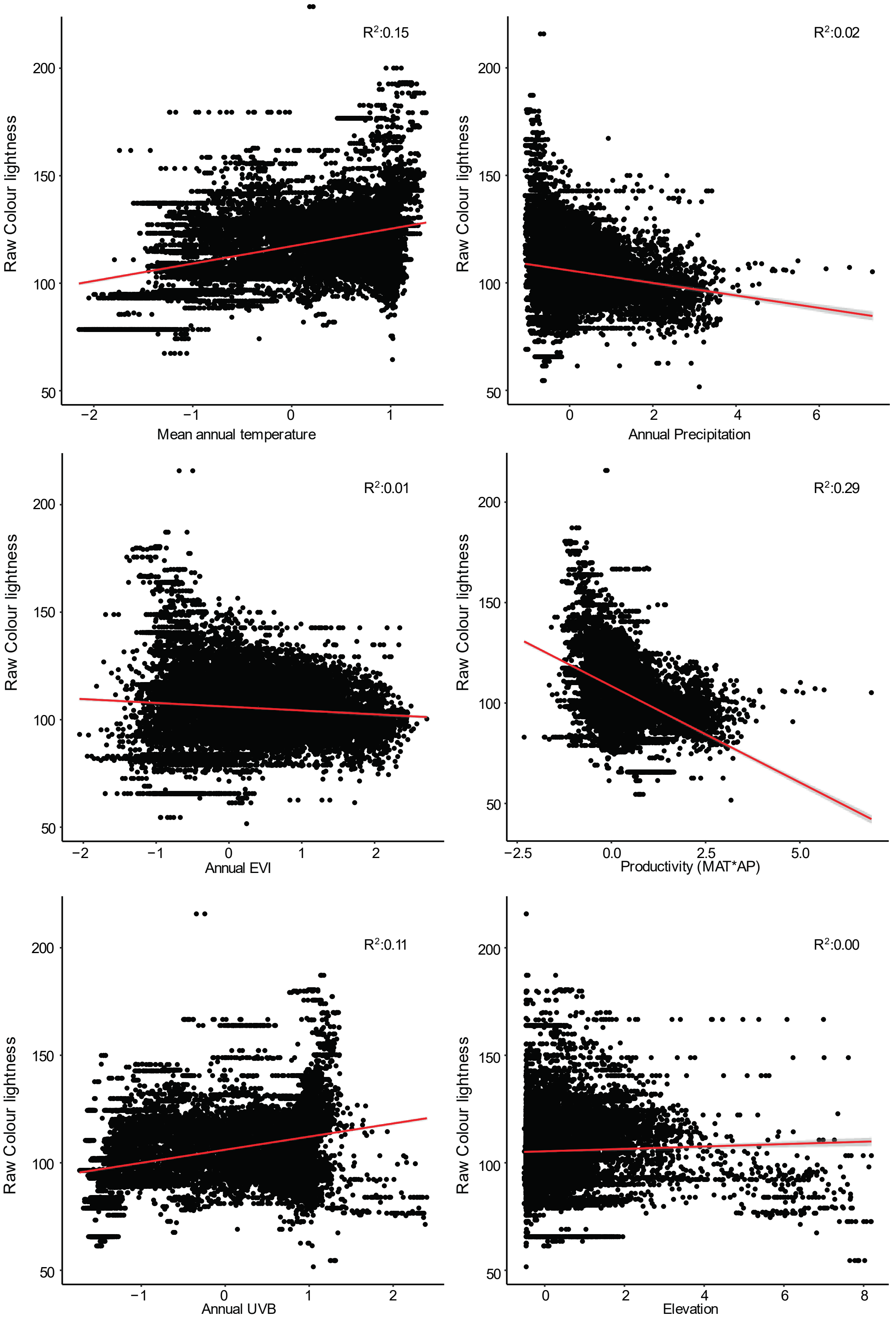

Supplementary Figure 1. Assemblage-level single linear regressions (n = 16,686 assemblages). Single linear regressions of assemblage level colour lightness and environmental variables (Mean Annual Temperature (MAT, slope ± SE = 8.08 ± 0.15, P < 10^{-16}), Annual Precipitation (AP, slope ± SE = –2.91 ± 0.144, P < 10^{-16}), Annual enhanced

vegetation index (EVI, slope ± SE = –1.77 ± 0.15, P = < 10^{-16}), Productivity (MAT*AP, slope ± SE = –7.77 ± 0.25, P = < 10^{-16}), Annual UVB radiation (slope ± SE = 6.10 ± 0.13, P < 10^{-16}) and Elevation (slope ± SE = 0.55 ± 0.12, P = 6.92 × 10^{-06})). All environmental variables are z-scaled. Regression lines are coloured red. Colour lightness ranges from 0 (black) to 255 (absolute white).

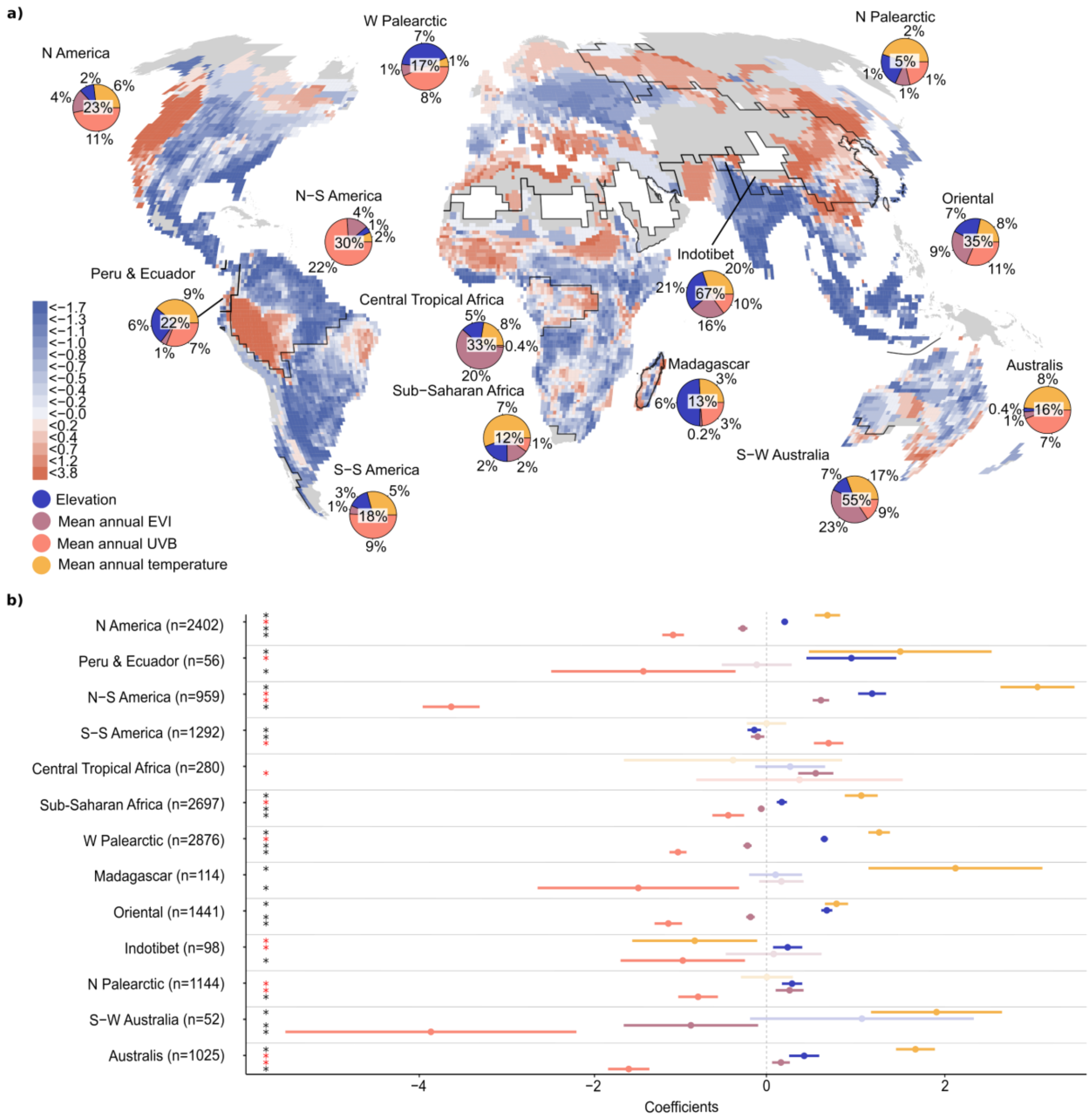

Supplementary Figure 2. Environmental drivers of colour lightness diversity.
a Geographical pattern in the variation of raw colour lightness across anuran assemblages (n = 14,436 assemblages; Mollweide projection). Blue colours display low and red colours display high colour lightness diversity (i.e., lower/higher than random). Pie charts in the inset of **a** show the independent contribution of environmental predictors (slices) as well as the overall R^2 (value in the circle centre) per biogeographic realm. For areas in grey, no data were available. **b** Coefficient means and 95% confidence intervals of the interaction effects of environmental predictors (z-scaled) and biogeographical realm on the raw colour lightness of anuran assemblages (overall $R^2 = 0.29$). Significant effects (no overlap with zero) are highlighted by an asterisk. Black asterisks indicate support for colour-based thermoregulation, pathogen resistance or UVB protection, whereas red asterisks indicate a converse trend. Only assemblages of biogeographic realms with more than 50 assemblages are considered. Shaded colours show non-significant correlations. (**MAT**:N Amerika $P < 10^{-16}$,Peru & Ecuador $P = 5.05 \times 10^{-03}$,N-S Amerika $P < 10^{-16}$, S-S America $P = 7.60 \times 10^{-01}$,Central Tropical Africa $P = 5.12 \times 10^{-01}$,Sub-Saharan Africa $P < 10^{-16}$, W Palearctic $P < 10^{-16}$, Madagascar P = 2.81

× $10^{–05}$, Oriental P < $10^{–16}$, Indotibet P = 1.89 × $10^{–02}$, N Palearctic P = 8.35 × $10^{–01}$, S-W Australia P = 5.88 × $10^{–07}$, Australis P < $10^{–16}$; **Annual EVI**: N Amerika P < $10^{–16}$, Peru & Ecuador P = 4.70 × $10^{–01}$, N-S Amerika P < $10^{–16}$, S-S America P = 4.38 × $10^{–04}$, Central Tropical Africa P = 2.84 × $10^{–07}$, Sub-Saharan Africa P = 2.00 × $10^{–06}$, W Palearctic P = 1.57 × $10^{–25}$, Madagascar P = 2.98 × $10^{–01}$, Oriental P < $10^{–16}$, Indotibet P = 8.68 × $10^{–01}$, N Palearctic P = 4.98 × $10^{–03}$, S-W Australia P = 2.17 × $10^{–02}$, Australis P = 1.25 × $10^{–02}$; **Elevation:** N Amerika P < $10^{–16}$, Peru & Ecuador P = 3.63 × $10^{–04}$, N-S Amerika P < $10^{–16}$, S-S America P = 8.57 × $10^{–06}$, Central Tropical Africa P = 2.50 × $10^{–01}$, Sub-Saharan Africa P = 2.88 × $10^{–06}$, W Palearctic P < $10^{–16}$, Madagascar P = 6.59 × $10^{–01}$, Oriental P < $10^{–16}$, Indotibet P = 1.54 × $10^{–02}$, N Palearctic P = 1.58 × $10^{–05}$, S-W Australia P = 1.06 × $10^{–01}$, Australis P = 6.63 × $10^{–06}$; **Annual UVB:** N Amerika P < $10^{–16}$, Peru & Ecuador P = 7.25 × $10^{–03}$, N-S Amerika P < $10^{–16}$, S-S America P = 4.55 × $10^{–15}$, Central Tropical Africa P = 5.71 × $10^{–01}$, Sub-Saharan Africa P = 3.28 × $10^{–07}$, W Palearctic P < $10^{–16}$, Madagascar P = 1.06 × $10^{–02}$, Oriental P = 1.22 × $10^{–46}$, Indotibet P = 6.23 × $10^{–03}$, N Palearctic P = 1.47 × $10^{–12}$, S-W Australia P = 5.11 × $10^{–06}$, Australis P < $10^{–16}$.

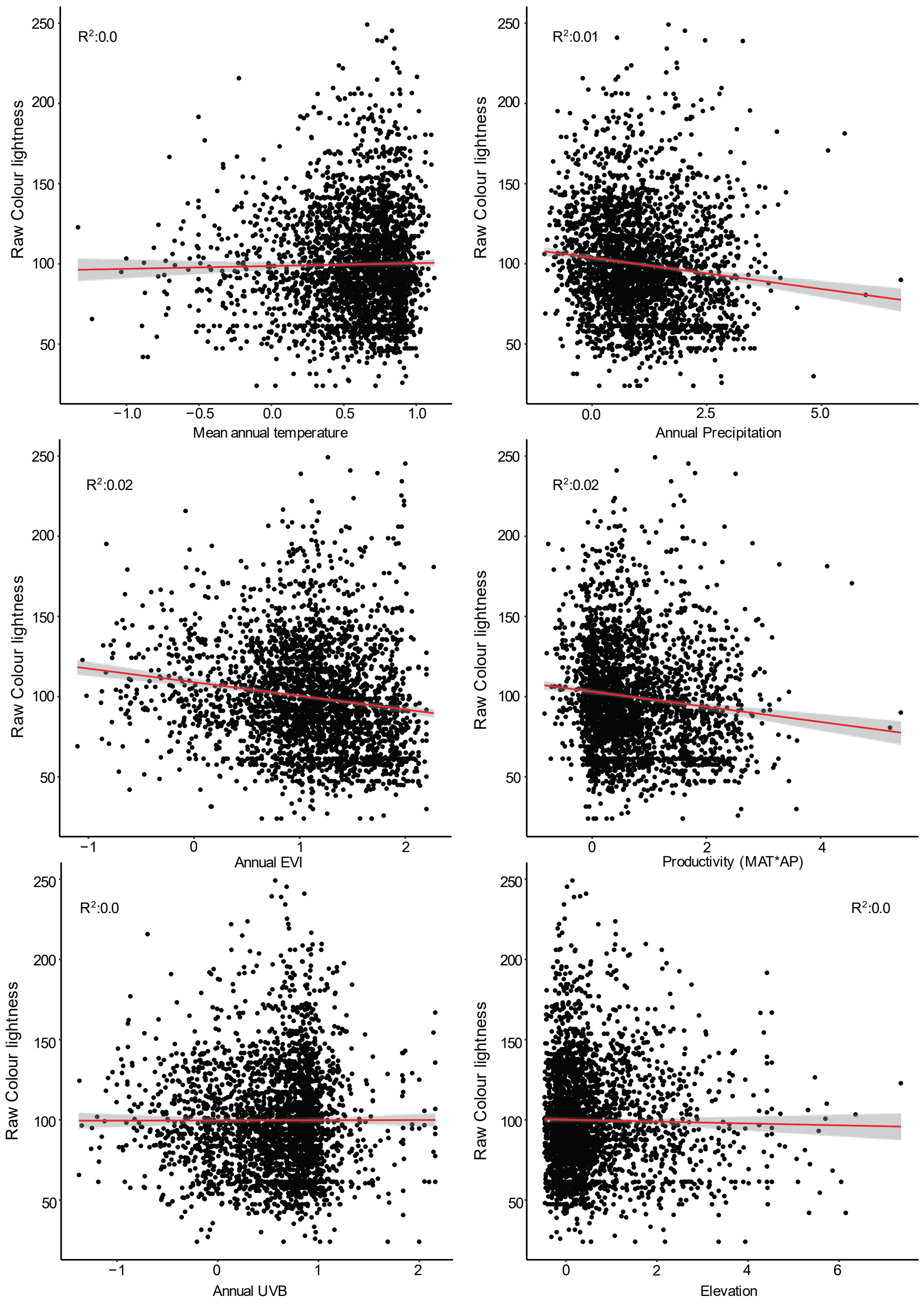

Supplementary Figure 3. Species level single linear regressions (n = 2984 species). Single linear regressions of species-level colour lightness and environmental variables (Mean Annual Temperature (MAT, slope ± SE = 1.76 ± 1.83, P = 0.336), Annual Precipitation (AP, slope ± SE = –3.88 ± 0.64, P = 1.08 × 10^{-09}), Annual enhanced vegetation index (EVI, slope ± SE = –8.51 ± 1.04, P < 10^{-16}), Productivity (MAT*AP, slope ± SE = –7.78 ± 2.30, P = 7.22 ×

10^{-04}), Annual UVB radiation (slope ± SE = 0.12 ± 1.22, P = 9.22 × 10^{-1}) and Elevation (slope ± SE = –0.59 ± 0.61, P = 3.48 × 10^{-1})). All environmental variables are z-scaled. Regression lines are coloured red. Colour lightness ranges from 0 (black) to 255 (absolute white). In these linear regressions, species belonging to families with fewer than 10 species were excluded.

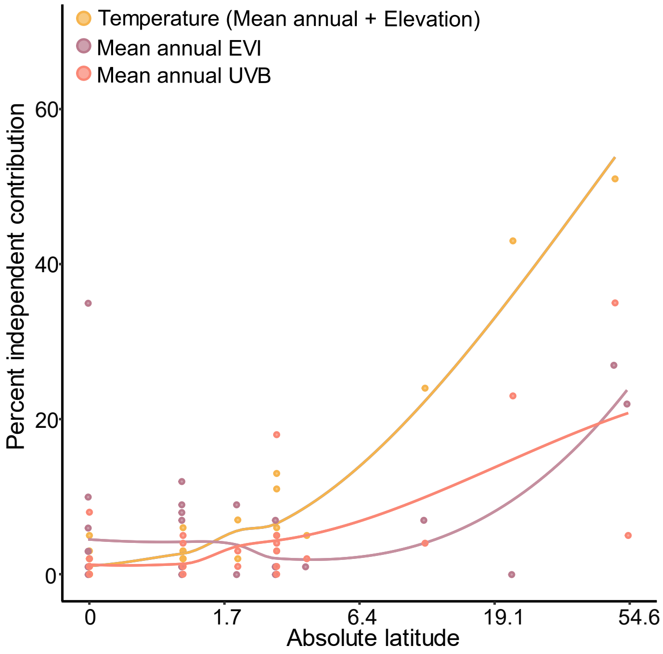

Supplementary Figure 4. Independent contribution of environmental variables. The percentage of the total explained variance in mean annual temperature, mean annual EVI (productivity) and mean annual UVB and the log+1 transformed latitudinal distribution centre of each family (absolute values averaged across all species) (n = 35 families). X-axis labels provide original values.

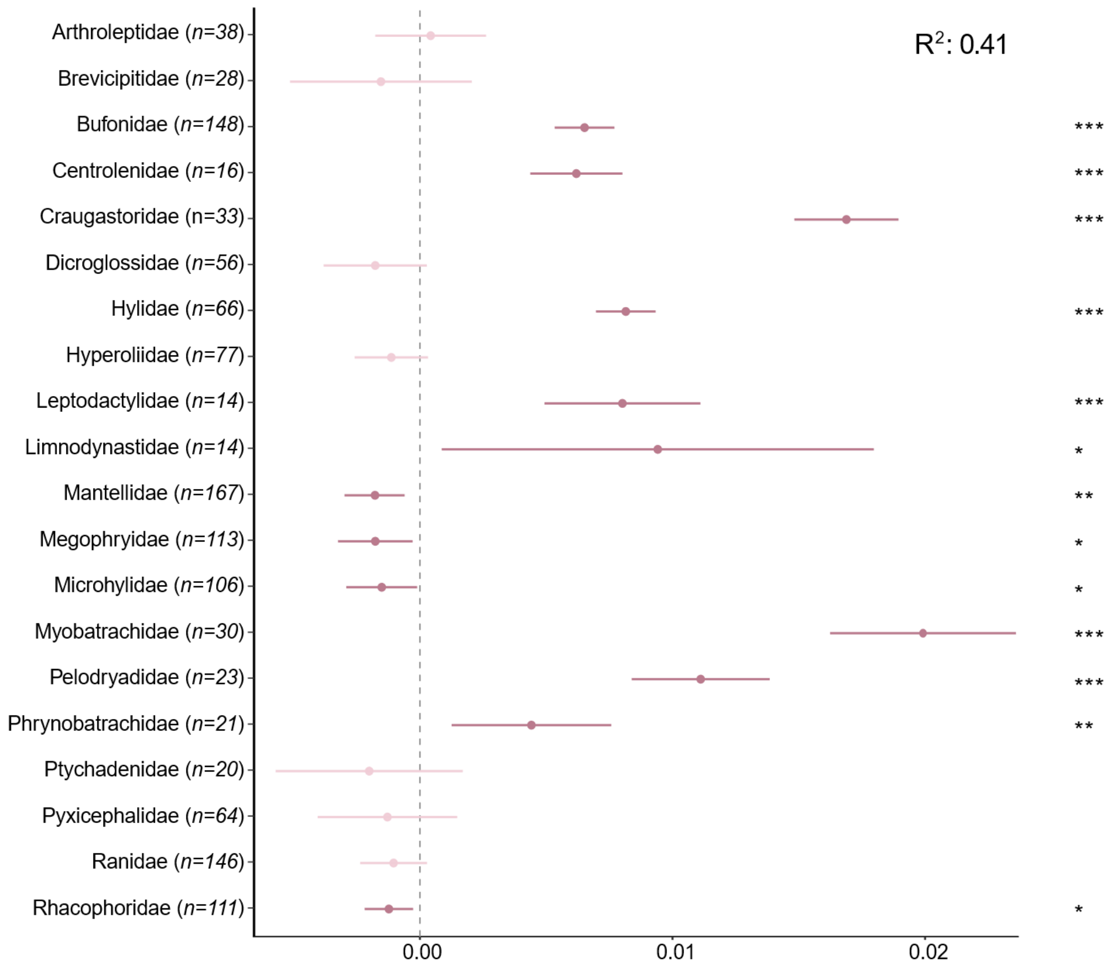

Supplementary Figure 5. The impact of colour-based pathogen resistance on severity of chytridiomycosis (n = 1,291 species). Coloured bars indicate the 95% confidence interval, and dots indicate the mean standardised effect size of a single regression (LM) of the interaction between (raw) colour lightness and productivity (Annual EVI) on chytridiomycosis severity[31] for anuran families (>9 species). According to the predictions of the Gloger's rule lighter-coloured species in regions with a higher productivity (higher pathogen pressure) should be more susceptible to fungal or bacterial pathogens. Hence the interaction of both high colour lightness and EVI should positively affect severity Black asterisks indicate significance levels (*** = 0.001, ** = 0.01, * = 0.05). Shaded colours show non-significant correlations. For models including all families and effects of colour and productivity on severity see Supplementary Tables 6-8. Arthroleptidae $P = 7.02 \times 10^{-1}$, Brevicipitidae $P = 4.00 \times 10^{-1}$, Bufonidae $P < 10^{-16}$, Centrolenidae: 4.06×10^{-11}, Craugastoridae $P < 10^{-16}$, Dicroglossidae $P = 9.02 \times 10^{-02}$, Hylidae $P < 10^{-16}$, Hyperoliidae 1.27×10^{-01}, Leptodactylidae $P = 4.01 \times 10^{-07}$, Limnodynastidae $P = 3.10 \times 10^{-02}$, Mantellidae $P = 3.17 \times 10^{-03}$, Megophryidae $P = 1.92 \times 10^{-02}$, Microhylidae $P = 3.49 \times 10^{-02}$, Myobatrachidae $P < 10^{-16}$, Pelodryadidae $P = 3.24 \times 10^{-15}$, Phrynobatrachidae $P = 6.23 \times 10^{-03}$, Ptychadenidae $P = 2.88 \times 10^{-01}$, Pyxicephalidae $P = 3.62 \times 10^{-01}$, Ranidae $P = 1.23 \times 10^{-01}$, Rhacophoridae $P = 1.23 \times 10^{-02}$.

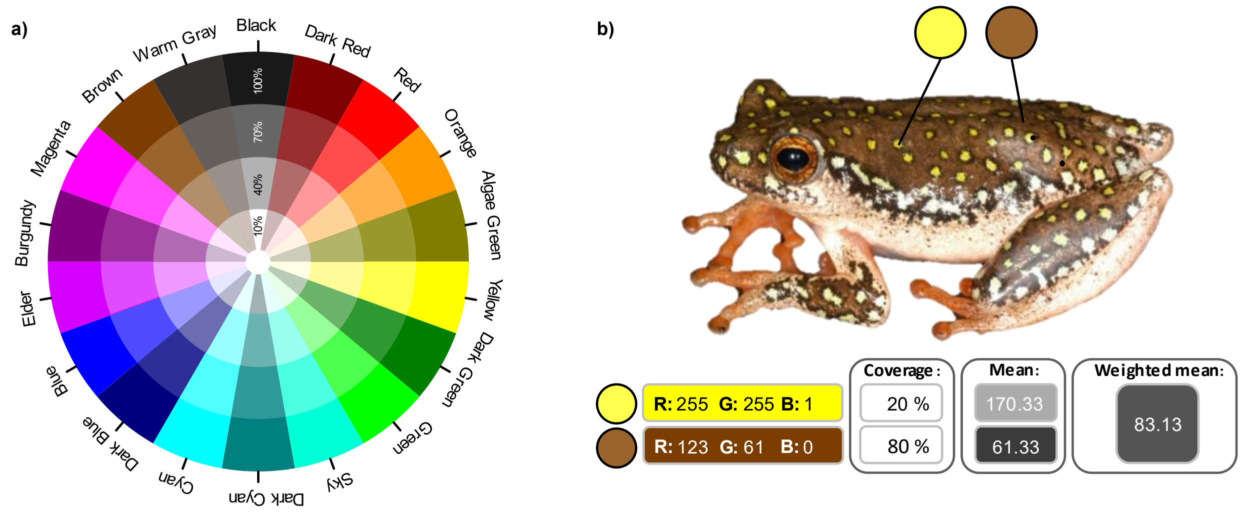

Supplementary Figure 6. Colour wheel and example of the procedure for estimating colour lightness. a The wheel comprises 18 rays, each consisting of four colours with different *Saturation* levels (S: 10, 40, 70, 100%). Six rays are based on colours that divide the *Hue* range into equally wide classes (H: 60, 120, 180, 240, 300, 360°). Another six rays are based on the same colours with a *Value* of 50 instead of 100 (e.g., dark green instead of green). Three rays are based on the complementary colours cyan, yellow, and magenta; and finally, three rays are based on black, warm grey and brown. The colour wheel was developed by SP and inspired by Bishop et al.[22]. The corresponding RGB values for each colour shown in the colour wheel are provided in Supplementary Table 3. The selection of colours is representative for the colour of the spectrum of light visual to humans but needed to incorporate narrow-ranging yet clearly distinguishable colours such as yellow and mixed colours such as brown and black. **b** Example calculation of dorsal coverage weighted mean colour lightness for *Hyperolius viridiflavus* (copyrights for the image are held by Alan Channing).

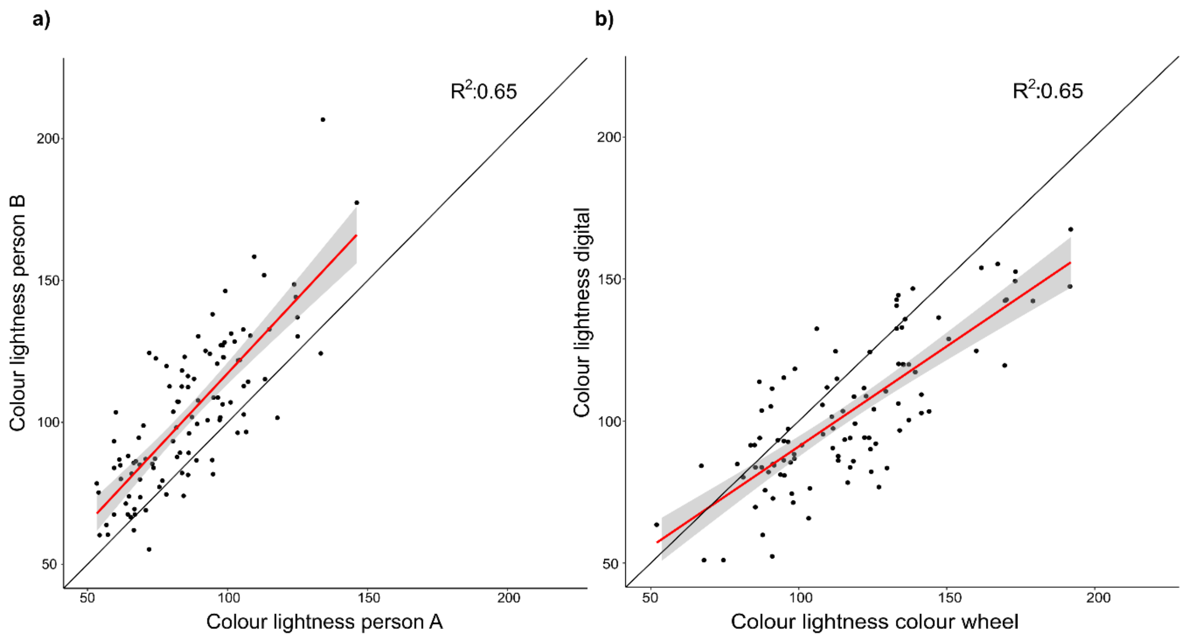

Supplementary Figure 7. Colour lightness estimates from two different persons and methods. a Linear regression of the colour lightness of Australian frogs and toads assessed by two persons. **b** Linear regression of colour lightness estimated assessed with the colour wheel and a digital probing approach. The latter estimates were calculated as the weighted mean RGB of distinct colours probed using ImageJ (Roberts et al. 2022). Mean RGB values were weighted based on the surface coverage of the corresponding colour of the dorsal body part. High values of colour lightness indicate lighter coloured species. Colour lightness ranges from 0 (black) to 255 (absolute white). Red and grey shaded lines indicate the regression and confidence interval, respectively. The black lines indicate the 1:1 lines. (**a**) slope ± SE = 0.60 ± 0.04, $P < 10^{-16}$ (n = 107 species); **b**) slope ± SE = 0.86 ± 0.06, $P < 10^{-16}$ (n = 107 species).

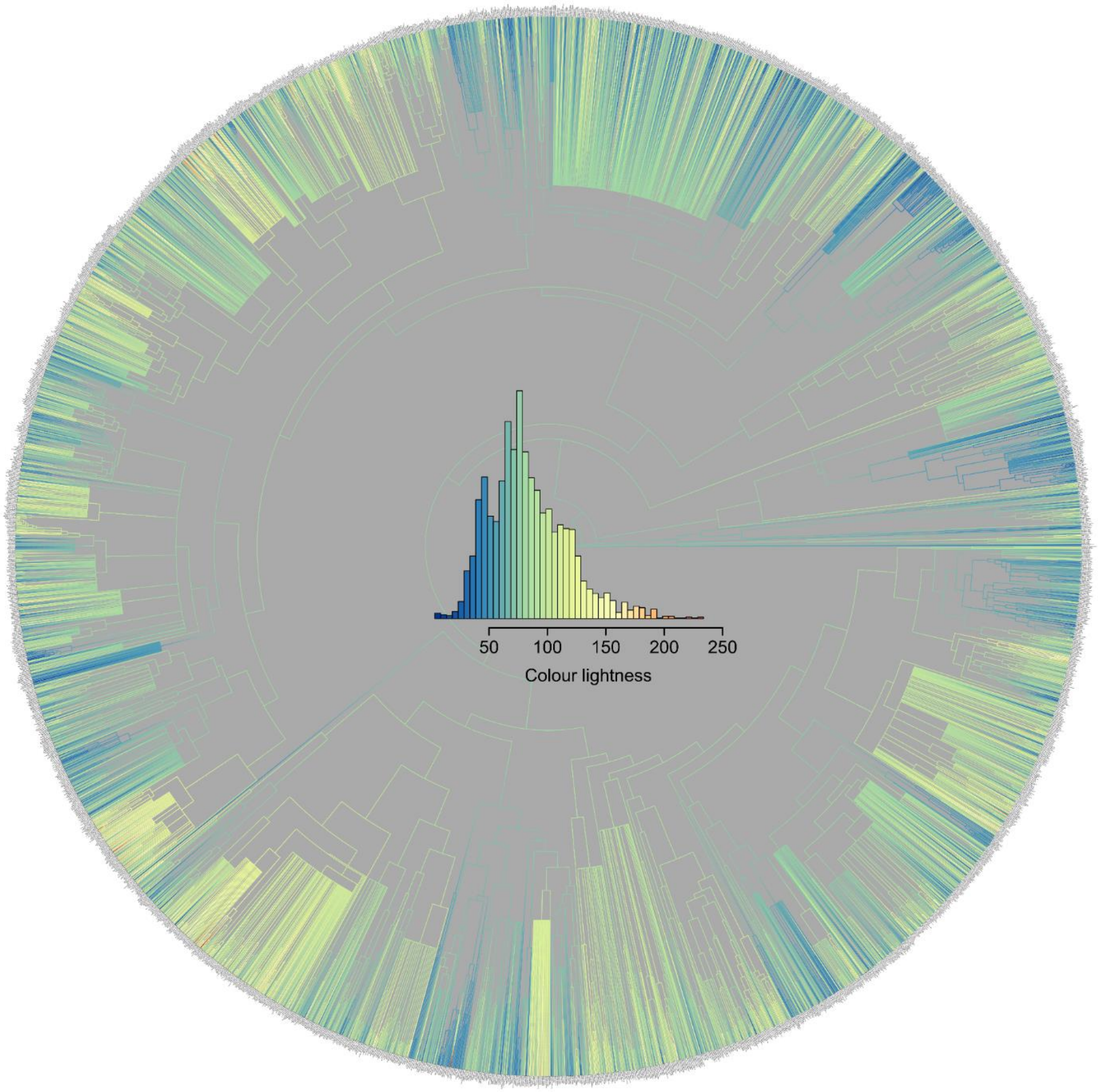

Supplementary Figure 8. Ancestral reconstruction of colour lightness variation across the phylogeny of 3,059 anuran species. Blue values indicate low colour lightness and red colours indicate high colour lightness of anuran species.

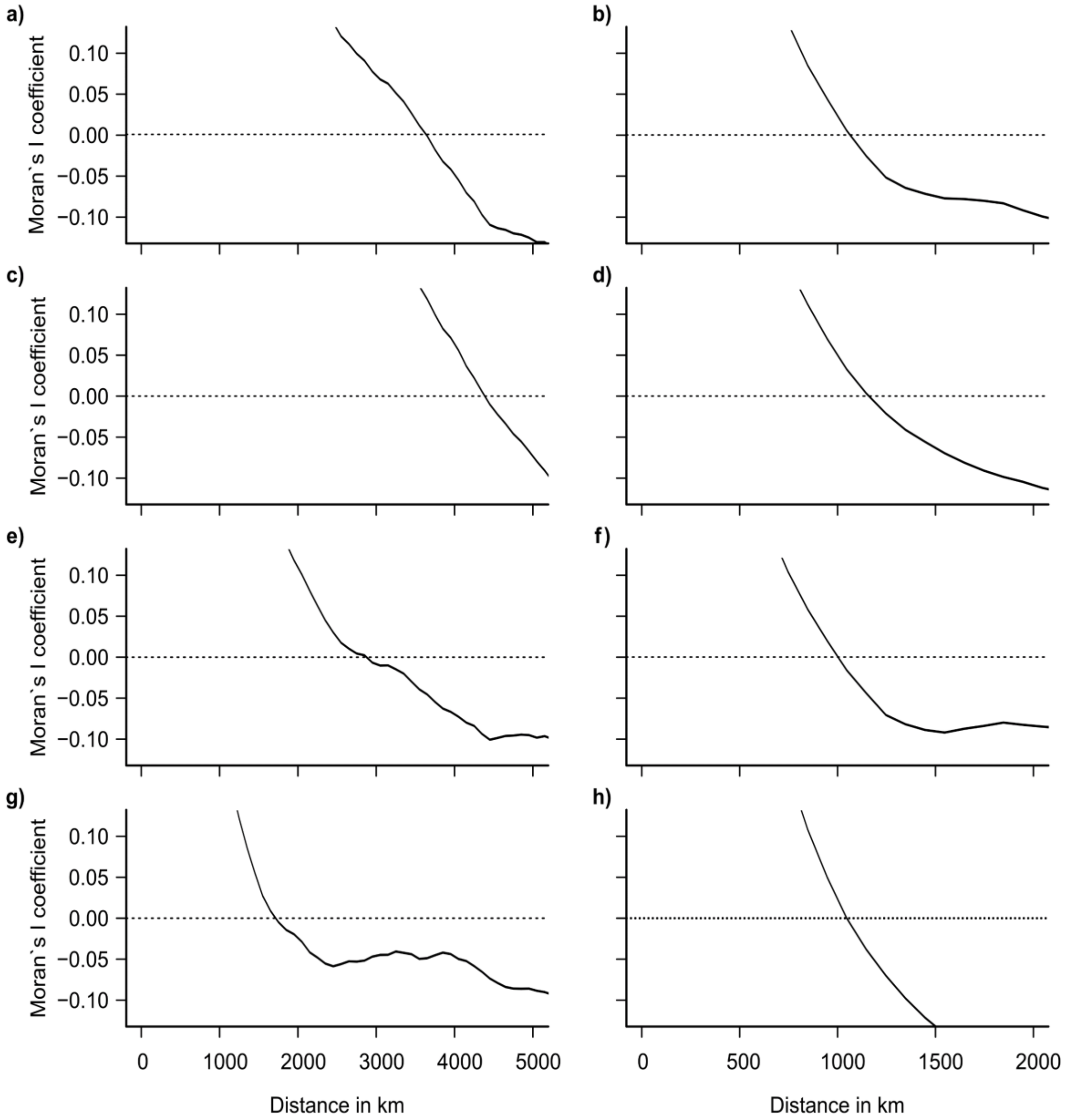

Supplementary Figure 9. Correlograms of multiple linear models at the assemblage level (n = 16,686 assemblages). a LM of raw colour lightness **b** GAM of raw colour lightness, **c** LM of the phylogenetic component, **d**, GAM of the phylogenetic component, **e** LM of the species-specific component, **f** GAM of the species-specific component**, g** LM of the functional diversity **h**, GAM of the functional diversity. The dashed line (the zero-line) indicates at what distance (in km) the variables are spatially independent from each other when the regression line crosses the dashed line. Results of the corresponding models are shown in Table 1.

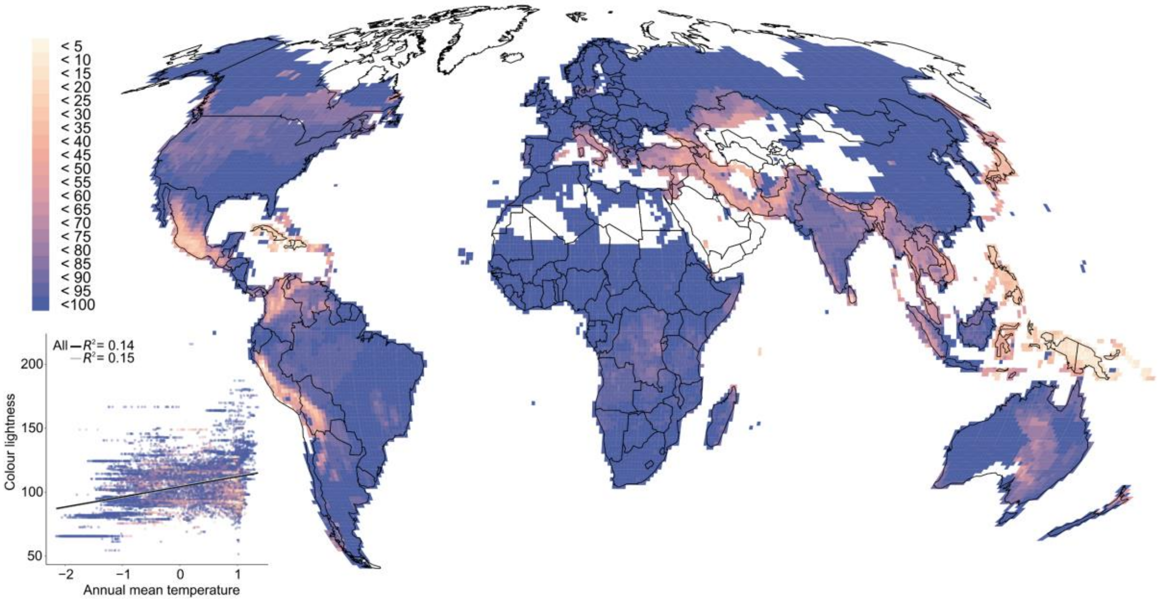

Supplementary Figure 10. Spatial coverage of colour lightness data for anuran assemblages. Colours in the scatterplot and map indicate the percentage of species per assemblage for which colour lightness data were available relative to the total number of species (n = 17,170 assemblages; map in Mollweide projection). Note that our decision to remove assemblages with a colour data coverage of less than 33% did not change the overall trends, for instance, in the relationship between colour lightness and annual mean temperature, but slightly reduced the scatter, as indicated by a higher coefficient of determination (all: slope ± SE = 7.89 ± 0.15, $P < 10^{-16}$; >33%: slope ± SE = 8.08 ± 0.15, $P < 10^{-16}$). Colour scale intervals follow an equal-distance classification ranging from high (plum) to low (light beige).
